# The multivariable prognostic models for severe complications after heart valve surgery

**DOI:** 10.1186/s12872-021-02268-z

**Published:** 2021-10-11

**Authors:** Yunqi Liu, Jiefei Xiao, Xiaoying Duan, Xingwei Lu, Xin Gong, Jiantao Chen, Mai Xiong, Shengli Yin, Xiaobo Guo, Zhongkai Wu

**Affiliations:** 1 Department of Cardiac Surgery, The First Affiliated Hospital of Sun Yat-Sen University, No.58, Zhongshan Road II, Guangzhou, 510080 China; 2 NCH Key Laboratory of Assisted Circulation, Sun Yat-Sen University, Guangzhou, 510080 China; 3 Department of Extracorporeal Circulation, The First Affiliated Hospital, Sun Yat-Sen University, Guangzhou, 510080 Guangdong China; 4 Department of Statistical Science, School of Mathematics, Sun Yat-Sen University, Guangzhou, China; 5 Southern China Center for Statistical Science, Sun Yat-Sen University, Guangzhou, 510275 China; 6 Department of Emergency, the Eighth Affiliated Hospital of Sun Yat-sen University, Shenzhen, 518000 China

**Keywords:** Multivariable prognostic model, Heart valve surgery, Low cardiac output syndrome, Acute kidney injury, Multiple organ dysfunction syndrome

## Abstract

**Background:**

To provide multivariable prognostic models for severe complications prediction after heart valve surgery, including low cardiac output syndrome (LCOS), acute kidney injury requiring hemodialysis (AKI-rH) and multiple organ dysfunction syndrome (MODS).

**Methods:**

We developed multivariate logistic regression models to predict severe complications after heart valve surgery using 930 patients collected retrospectively from the first affiliated hospital of Sun Yat-Sen University from January 2014 to December 2015. The validation was conducted using a retrospective dataset of 713 patients from the same hospital from January 2016 to March 2017. We considered two kinds of prognostic models: the PRF models which were built by using the preoperative risk factors only, and the PIRF models which were built by using both of the preoperative and intraoperative risk factors. The least absolute shrinkage selector operator was used for developing the models. We assessed and compared the discriminative abilities for both of the PRF and PIRF models via the receiver operating characteristic (ROC) curve.

**Results:**

Compared with the PRF models, the PIRF modes selected additional intraoperative factors, such as auxiliary cardiopulmonary bypass time and combined tricuspid valve replacement. Area under the ROC curves (AUCs) of PRF models for predicting LCOS, AKI-rH and MODS are 0.565 (0.466, 0.664), 0.688 (0.62, 0.757) and 0.657 (0.563, 0.751), respectively. As a comparison, the AUCs of the PIRF models for predicting LOCS, AKI-rH and MODS are 0.821 (0.747, 0.896), 0.78 (0.717, 0.843) and 0.774 (0.7, 0.847), respectively.

**Conclusions:**

Adding the intraoperative factors can increase the predictive power of the prognostic models for severe complications prediction after heart valve surgery.

## Backgrounds

Heart valve disease (HVD) is a common cardiosurgery disease, mainly including rheumatic, degenerative, ischemic and myxoid valvular disease [1]. The 30-day mortality after heart valve surgery is about 4–6%, nearly two-fold higher than coronary artery bypass graft (CABG) [2–4]. The morbidity of HVD also increases with increasing aging population [5, 6].

In the past 30 years, there are numerous conventional prognostic models to predict in-hospital mortality for patients who underwent cardiac surgery, such as the European System for Cardiac Operation Risk Evaluation (EuroSCORE), Quality Measurement and Management Initiative (QMMI), Northern New England Cardiovascular Disease Study Group (NNECDSG), New York’s Cardiac Surgery Reporting System (NYCSRE) and Society of Thoracic Surgeons (STS) score [3, 7–12]. However, these prognostic models mainly predict postoperative mortality for CABG by preoperative factors, neither heart valve surgery nor severe complications prediction is concerned.

As the clinical observations and researches show, the major causes of mortality were severe complications after heart valve surgery, such as low cardiac output syndrome (LCOS), acute kidney injury requiring hemodialysis (AKI-rH) and multiple organ dysfunction syndrome (MODS) [13–15]. These severe complications not only prolong hospital stay, but also increase the hospitalization expenses of patients. Meanwhile, some important intraoperative factors, especially cardiopulmonary bypass (CPB)-related factors significantly affect the complications and morbidity [16, 17].

Therefore, this study aims to provide a method considering both preoperative and intraoperative factors to predict severe complications for patients who underwent heart valve surgery within 30 days. Further, to provide a thought for these conventional prognostic models to more accurately predict mortality.

## Methods

### Patients selection

This was a retrospective observational study of total 1643 adult patients who underwent heart valve surgery from January 2014 to March 2017 in the First Affiliated Hospital of Sun Yat-sen University, Guangzhou, China. The 930 patients (445 males, 485 females) admitted from January 2014 to December 2015 were used for model development. The other 713 patients (370 males, 343 females) admitted from January 2016 to March 2017 were used for model validation.

The inclusion criteria for patients selection should be adult patients older than 18 years, without history of any mechanical assistant due to organ failure.

The investigation complied with the principles of the Declaration of Helsinki and was approved by the human ethics committee of the First Affiliated Hospital of Sun Yat-sen University. Written informed consent forms were obtained from all patients.

### Data collection

The preoperative clinical data were collected from patients’ demographics, medical histories, results of essential laboratory tests and routine imaging examinations. The intraoperative clinical data were collected from surgical approaches, defibrillation frequency, aortic occlusion time (AOT) and auxiliary CPB time (ACPBT). The postoperative clinical data were collected from severe complications, mechanical assistant and discharge status.

All patients had a 30-day follow-up after cardiac valve surgery. The endpoints were the postoperative severe complications (LCOS, AKI-rH and MODS) within 30 days. Treatment principles of patients with cardiac valve disease were coincidences with international guidelines [18–21].

### The definitions of severe complications

LCOS: (1) cardiac index (CI) < 2 min m^2^ and systolic blood pressure (SBP) < 90 mmHg; (2) mixed venous oxygen saturation (SvO_2_) < 50% and arterial oxygen saturation (SaO_2_) minus SvO_2_ ≥ 30%; (3) metabolic acidosis: the base excess indicate (B.E.) <  − 4; (4) signs of tissue hypoperfusion; (5) the results of Swan-Ganz catheterization, pulse index contour cardiac output (PiCCO), and echocardiography [13, 22–25].

MODS: It’s a frequent complication of systemic inflammatory response syndrome, which presences of altered organ function in an acutely ill patients such that homeostasis cannot be maintained without intervention [26].

AKI-rH: (1) blood creatinine (BCr) ≥ 3 times baseline or BCr ≥ 354 mmol/l with the elevated level ≥ 44 mmol/l within 48 h; (2) oliguria: urine output less than 0.3 ml/kg/h for ≥ 24 h; (3) anuria for ≥ 12 h [27–29].

### Statistical analysis

Analyses were performed in R version 3.5.1.

**Fig. 1:**
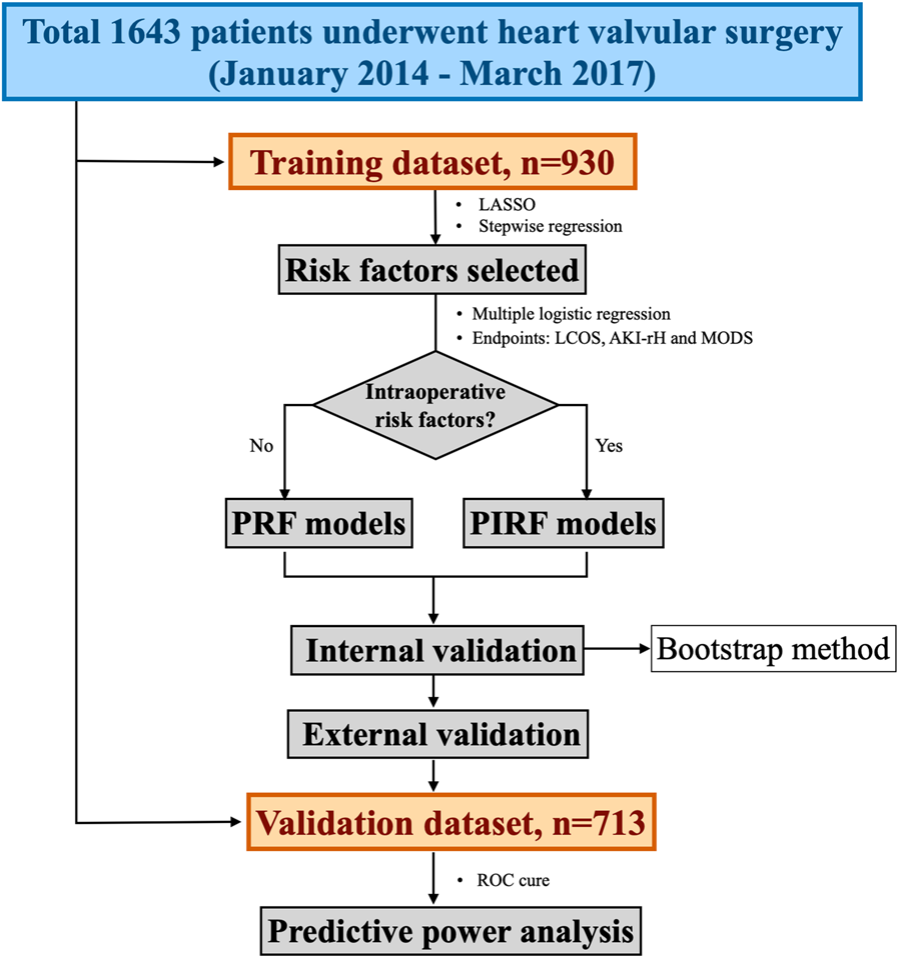
Establishment and validation of PRF and PIRF models

Two variable selection methods were respectively applied to build prognostic models: (1) preoperative variables were selected to build preoperative risk factors (PRF) models; (2) both preoperative and intraoperative variables were selected to build preoperative and intraoperative risk factors (PIRF) models (Fig. 1).

Compared the two prognostic models, we could conclude whether the predictive power improved when intraoperative risk factors added. Besides, considered a significant correlation might exist between preoperative and intraoperative risk factors in PIRF models, synchronous variable selection was performed among all related preoperative and intraoperative risk factors, rather than selecting separately. Using the least absolute shrinkage selector operator (LASSO) or stepwise regression analysis could effectively decrease the data dimensionality, further enhanced the predictive effect of the PIRF model. A multiple logistic regression was established to compare the selected risk factors between these two kinds of models which were respectively applied to predict the three endpoints (LCOS, AKI-rH and MODS).

During the evaluation period, internal and external validation were separately processed in these two kinds of models. In internal validation, bootstrap method with 1000 resampling was used to reduce overfitting of training dataset to obtain the internal evaluation results. In external validation, the prognostic models built by training dataset were applied for validation dataset to obtain the evaluation results. A multi-dimensional comparison included receiver operating characteristic curve (ROC) and the area under the ROC (AUC) of validation dataset was performed to estimate and compare the accuracy of PRF and PIRF models.

## Results

### Patients’ characteristics

The characteristics of training and validation datasets are listed in Table 1. Compared the two datasets, the morbidities of severe complications are LCOS (9.46% vs. 5.33%, *P* < 0.05), AKI-rH (4.48% vs. 7.29%, *P* < 0.05) and MODS (4.95% vs. 4.49%, *P* > 0.05), respectively.

**Table 1:** Patient characteristics

Characteristics	Training dataset	Validation dataset	*P*
	(n = 930)	(n = 713)	
*Demographics*			
Age (y)	47.91 ± 13.83	49.68 ± 15.00	0.001
Gender (female, No. %)	485(52.15%)	343(48.11%)	0.115
Height (cm)	160.73 ± 8.18	160.49 ± 10.69	0.939
Weight (kg)	54.66 ± 10.39	56.96 ± 11.95	< 0.01
BMI	21.08 ± 3.29	21.97 ± 3.62	< 0.01
BSA (m^2^)	6.87 ± 1.29	7.15 ± 1.49	< 0.01
Smoke (No. %)	166 (17.85%)	95(13.32%)	0.016
*Medical histories*			
CF (< 4 weeks, No. %)	601(64.62%)	421(59.05%)	0.024
Endocarditis (No. %)	88(9.46%)	120(16.83%)	< 0.01
Diabetes (No. %)	48(5.16%)	49(6.87%)	0.176
Hypertension (No. %)	122(13.12%)	129(18.09%)	0.007
Hepatitis (No. %)	78(8.39%)	23(3.23%)	< 0.01
Pulmonary disease (No. %)	78(8.39%)	30(4.21%)	0.001
Dialysis (No. %)	0(0.00%)	0(0.00%)	< 0.01
PVD (No. %)	0(0.00%)	0(0.00%)	< 0.01
Re-operation (No. %)	58(6.24%)	52(7.29%)	0.453
*Laboratory values*			
WBC (× 10^9^/l)	7.04 ± 2.46	7.22 ± 2.38	0.076
PLT (× 10^12^/l)	213.26 ± 66.44	217.74 ± 82.53	0.789
RBC (× 10^9^/l)	4.68 ± 0.70	4.58 ± 0.76	0.003
RBC-DW	0.14 ± 0.02	0.14 ± 0.03	< 0.001
< 0.12	3(0.32%)	1(0.14%)	
0.12–0.15	772(83.01%)	560(78.54%)	
> 0.15	148(15.91%)	151(21.18%)	
Hb (g/l)	133.78 ± 19.63	130.54 ± 21.53	0.001
ALT (u/l)	25.79 ± 33.6	26.94 ± 29.51	0.998
ALB (g/l)	42.29 ± 18.01	39.36 ± 4.92	< 0.01
TBil (mmol/l)	15.88 ± 10.39	16.52 ± 9.76	0.001
BUA (mg/l)	374.78 ± 122.51	423.22 ± 140.87	< 0.01
BUN (mmol/l)	6.12 ± 2.51	6.15 ± 2.93	0.249
< 2.9	24(2.58%)	22(3.09%)	
2.9–8.6	802(86.24%)	606(84.99%)	
> 8.6	95(10.22%)	84(11.78%)	
BCr (umol/l)	77.19 ± 27.07	86.11 ± 68.24	0.01
< 50	56(6.02%)	43(6.03%)	
50–115	808(86.88%)	609(85.41%)	
116–200	55(5.91%)	51(7.15%)	
> 200	3(0.32%)	9(1.26%)	
BUN/BCr	0.08 ± 0.04	0.08 ± 0.03	< 0.01
< 0.055 (No. %)	127(13.66%)	143(20.06%)	
0.055–0.075 (No. %)	308(33.12%)	250(35.06%)	
> 0.075 (No. %)	486(52.26%)	319(44.74%)	
CCr (ml/min/1.73m^2^, No.)	79.62 ± 34.28	76.17 ± 26.37	0.046
< 50 (No. %)	116(12.47%)	98(13.74%)	
50–80 (No. %)	396(42.58%)	338(47.41%)	
> 80 (No. %)	404(43.44%)	275(38.57%)	
APTT (secs.)	29.3 ± 5.72	31.74 ± 6.75	< 0.01
Fbg (g/l)	3.08 ± 1.16	3.08 ± 1.20	0.419
ESR (mm)	23.49 ± 20.45	26.16 ± 23.34	0.192
*ECG measurements*			
Atrial fibrillation (No. %)	389(41.83%)	235(32.96%)	< 0.01
*UCG measurements*			
LVD (mm)	54.51 ± 11.10	53.65 ± 10.97	0.220
EF (%)	62.71 ± 10.14	63.95 ± 9.93	0.005
> 50	777(83.55%)	612(85.83%)	
30–50	93(10%)	72(10.1%)	
< 30	4(0.43%)	1(0.14%)	
PASP (mmHg)	21.86 ± 27.54	45.59 ± 17.4	< 0.01
> 60	80(8.6%)	86(12.06%)	
30–60	310(33.33%)	380(53.3%)	
< 30	540(58.06%)	57(7.99%)	
*Intraoperative variables*			
AOT (min)	80.23 ± 34.70	90.0 ± 46.70	0.001
ACPBT (min)	37.7 ± 22.50	58.4 ± 40.20	< 0.01
*Defibrillation (freq.)*			0.351
< 1	773(83.12%)	605(84.85%)	
≥ 1	157(16.88%)	108(14.79%)	
*Surgical approaches*			
AVR (No. %)	378(40.65%)	293(41.09%)	0.894
MVR (No. %)	684(73.55%)	432(60.59%)	< 0.01
TVR (No. %)	33(3.55%)	31(4.35%)	0.483
MVP (No. %)	57(6.13%)	84(11.78%)	< 0.001
TVP (No. %)	298(32.04%)	303(42.5%)	< 0.001
CABG (No. %)	28(3.01%)	32(4.49%)	0.147
RFA (No. %)	27(2.9%)	33(4.63%)	0.086
Other cardiac surgery (No. %)	60(6.45%)	210(29.45%)	< 0.001
Non-cardiac surgery (No. %)	4(0.43%)	0(0.00%)	0.138
*Severe complications*			
LCOS (No. %)	88(9.46%)	38(5.33%)	0.002
AKI-rH (No. %)	45(4.84%)	52(7.29%)	0.047
MODS (No. %)	46(4.95%)	32(4.49%)	0.752
*Mechanical assistant*			
IABP /ECMO (No. %)	33(3.55%)	29(4.07%)	0.677
*Discharge status*			
Death (No. %)	61(6.56%)	47(6.59%)	1.000

The age in training dataset is younger than validation dataset (47.91 ± 13.83 vs. 49.68 ± 15 years, *P* < 0.05). The morbidities of preoperative pulmonary disease (PD) and hepatitis of training dataset were higher than that of validation dataset (PD: 8.39% vs. 4.21%, *P* < 0.05; hepatitis: 8.39% vs. 3.23%, *P* < 0.05). More patients had a previous history of endocarditis in validation dataset than training dataset (16.83% vs. 9.46%, *P* < 0.05). According to the results of echocardiography, preoperative eject functions (EF) of training and validation datasets are 62.71 ± 10.14% and 63.95 ± 9.93%, respectively. Besides, pulmonary artery systolic pressure (PASP) in validation dataset is higher than that in training dataset (45.59 ± 17.4 mmHg vs. 21.86 ± 27.54 mmHg, *P* < 0.05). Intraoperative AOT and ACPBT of training dataset are both shorter than those in validation dataset (AOT: 80.23 ± 34.7 min vs. 90.0 ± 46.7 min, *P* < 0.01; ACPBT: 37.7 ± 22.5 min vs. 58.4 ± 40.2 min, *P* < 0.01).

### Prognostic models for LCOS

**Fig. 2:**
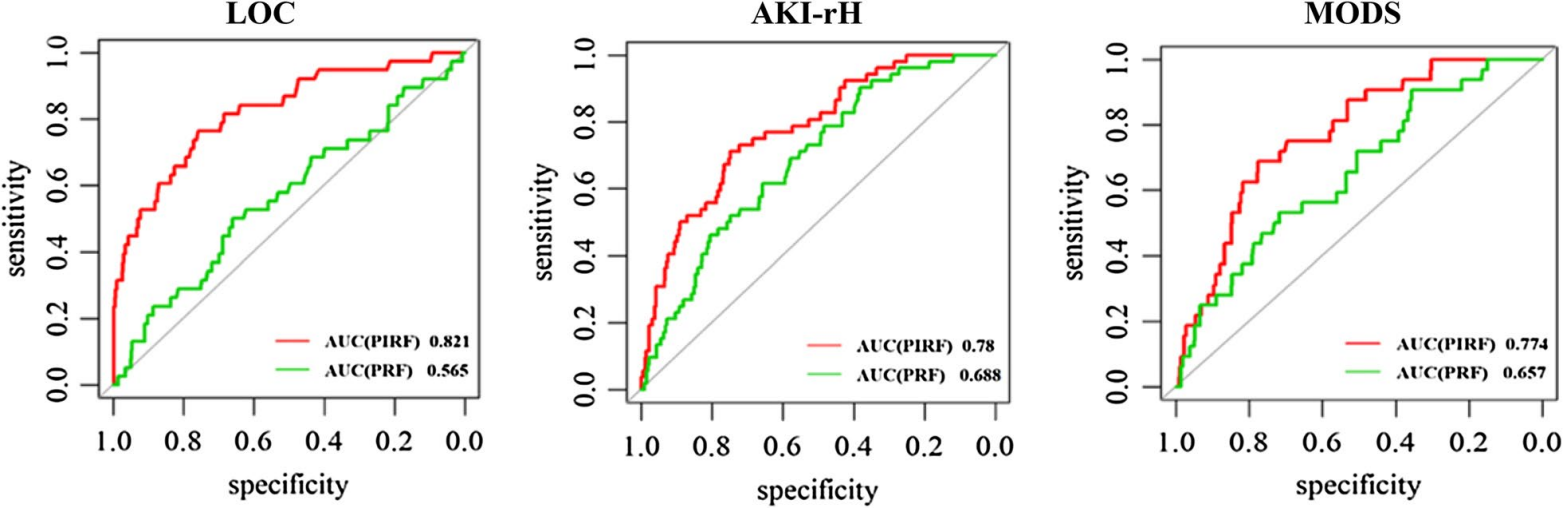
ROC curve for different complications in validation datasets

The PRF model for LCOS includes BCr (OR 1.85; 95% CI 0.95–3.59), creatinine clearance rate (CCr) (OR 0.46; 95% CI 0.32–0.67), hemoglobin (Hb) (OR 0.73; 95% CI 0.58–0.91), PAH (OR 1.34; 95% CI 0.96–1.86), and hypertension (OR 1.70; 95% CI 0.94–3.05) (Table 2). As a comparison, the PIRF model only includes CCr (OR 0.38; 95% CI 0.27–0.53) and ACPBT (OR 1.80; 95% CI 1.52–2.12).We applied both models to the validation dataset. The AUC of the PIRF model is 0.821 (0.747, 0.896), which is statistically higher (*P* < 0.01) than that 0.565 obtained in the PRF model (Fig. 2, Table 5).

**Table 2:** Prognostic models for LCOS in development dataset

Variables	PRF model (n = 930)			PIRF model (n = 930)		
	β	OR (95% CI)	*P*	β	OR (95% CI)	*P*
Intercept	− 2.4909	0.08	0.015	− 0.3645	0.69	0.311
BCr	0.6152	1.85 (0.95–3.59)	0.068			
CCr	− 0.7756	0.46 (0.32–0.67)	< 0.01	− 0.9801	0.38 (0.27–0.53)	< 0.01
Hb	− 0.3191	0.73 (0.58–0.91)	0.006			
PASP	0.2929	1.34 (0.96–1.86)	0.082			
Hypertension	0.5281	1.70 (0.94–3.05)	0.079			
ACPBT				0.5855	1.80 (1.52–2.12)	< 0.01

### Prognostic models for AKI-rH

The PRF model for AKI-rH includes CCr (OR 0.33; 95% CI 0.21–0.52), red blood cell distribution width (RBC-DW) (OR 2.59; 95% CI 1.31–5.13) and total bilirubin (TBil) (OR 1.51; 95% CI 1.20–1.90) (Table 3). As a comparison, the PIRF model includes CCr (OR 0.36; 95% CI 0.22–0.57), RBC-DW (OR 2.19; 95% CI 1.08–4.43), TBil (OR 1.52; 95% CI 1.21–1.92) and ACPBT (OR 1.50; 95% CI 1.23–1.82). We applied both models to the validation dataset. The AUC of the PIRF model is 0.78 (0.717, 0.843), which is statistically higher (*P* < 0.01) than that 0.688 obtained in the PRF model (Fig. 2, Table 5).

**Table 3:** Prognostic models for AKI-rH in development dataset

Variables	PRF model (n = 930)			PIRF model (n = 930)		
	β	OR (95%CI)	*P*	β	OR (95% CI)	*P*
Intercept	− 2.9392	0.05	0.002	− 2.8605	0.06	0.004
CCr	− 1.1041	0.33 (0.21–0.52)	< 0.01	− 1.0247	0.36 (0.22–0.57)	< 0.01
RBC-DW	0.9530	2.59 (1.31–5.13)	0.006	0.7835	2.19 (1.08–4.43)	0.030
TBil	0.4093	1.51 (1.20–1.90)	0.001	0.4206	1.52 (1.21–1.92)	< 0.01
ACPBT				0.4042	1.50 (1.23–1.82)	< 0.01

### Prognostic models for MODS

The PRF model for MODS includes CCr (OR 0.28; 95% CI 0.18–0.45), BUN/BCr (OR 1.81; 95% CI 1.11–2.95), Hb (OR 0.74; 95% CI 0.55–1.01), heart failure history (OR 1.84; 95% CI 0.82–4.16) and PD (OR 3.33; 95% CI 1.55–7.16) (Table 4). As a comparison, the PIRF model includes CCr (OR 0.29; 95% CI 0.17–0.48), BUN/BCr (OR 1.86; 95% CI 1.1–3.14), CF (< 4 weeks) (OR 1.95; 95% CI 0.83–4.58), PD (OR 4.69; 95% CI 2.10–10.47), ACPBT (OR 1.71; 95% CI 1.41–2.09) and combined with tricuspid valve replacement (cTVR) (OR 3.69; 95% CI 1.16–11.47). We applied both models to the validation dataset. The AUC of the PIRF model is 0.774 (0.70, 0.847), which is statistically higher (*P* < 0.01) than that 0.657 obtained in the PRF model (Fig. 2, Table 5).

**Table 4:** Prognostic models for MODS in development dataset

Variables	PRF model (n = 930)			PIRF model (n = 930)		
	β	OR (95% CI)	*P*	β	OR (95% CI)	*P*
	− 2.5690	0.08	0.002	− 3.0533	0.05	0.001
CCr	− 1.2645	0.28 (0.18–0.45)	< 0.01	− 1.2457	0.29 (0.17–0.48)	< 0.01
BUN/BCr	0.5907	1.81 (1.11–2.95)	0.018	0.6219	1.86 (1.10–3.14)	0.020
Hb	− 0.296	0.74 (0.55–1.01)	0.057			
CF	0.6110	1.84 (0.82–4.16)	0.141	0.6660	1.95 (0.83–4.58)	0.127
PD	1.2038	3.33 (1.55–7.16)	0.002	1.5459	4.69 (2.10–10.47)	< 0.01
ACPBT				0.5381	1.71 (1.41–2.09)	< 0.01
cTVR				1.3049	3.69 (1.16–11.47)	0.027

**Table 5:** Comparisons of PRF and PIRF models for three complications in validation dataset

Complications	AUC		
	PRF model (n = 713)	PIRF model (n = 713)	*P*
LCOS	0.565 (0.466, 0.664)	0.821 (0.747, 0.896)	< 0.01
AKI-rH	0.688 (0.62, 0.757)	0.78 (0.717, 0.843)	< 0.01
MODS	0.657 (0.563, 0.751)	0.774 (0.7, 0.847)	0.003

## Discussions

The postoperative mortality of cardiac surgery obviously declines to 1–2%, but the morbidity of postoperative severe complications (LCOS, AKI-rH and MODS) still remains high, caused by surgical trauma, CPB-related injury, ischemia–reperfusion, endotoxemia, and blood transfusion repeatedly [24, 30]. These complications will prolong hospital stay and increase hospitalization costs [31, 32] .

Postoperative LCOS is one of the most serious complications and major cause to high mortality [13]. Around 70% of postoperative cardiac surgery patients have signs of ventricular systolic and diastolic dysfunction. Brain, liver, and kidney failure are common consequences of LCOS, eventually leading to MODS. In this study, we re-selected and re-determined relative risk variables by adding intraoperative factors and found that only CCr and ACPBT were the independent risk factors for postoperative LCOS. Manjula et al. also suggested preoperative renal failure (OR 4.9) was the most influential predictor for postoperative LCOS in the isolated aortic valve surgery [33]. Auxiliary cardiopulmonary bypass is a important way to repay myocardial ischemic oxygen debt with total bypass [34, 35]. But the clear advice on what is the optimal ACPBT is still not available from the scientific literature [36, 37]. ACPBT is often empirically controlled at the 20–30% of AOT, prolonged ACPBT could also decrease the difficulty of weaning from CPB [38, 39].

Postoperative AKI-rH is another cause to high mortality. More than 35% of patients before heart valve surgery have a previous history of chronic kidney disease, it is also a significant independent predictor of postoperative short-and long-term mortality [14, 40]. Approximately 40–50% of patients underwent heart valve surgery have acute kidney injury (AKI) attributed to ischemia–reperfusion injury during surgery, especially for the elder, diabetic and coronary artery disease (CAD) patients [40, 41]. The incidence of AKI-rH is nearly 1–3%, it is on the rise due to the increase of surgery complexity and tends to cause end-stage renal disease (ESRD) [4, 29, 42]. Compared the risk factors for postoperative AKI-rH between PRF and PIRF model, we found ACPBT was added additionally in the PIRF model besides the perioperative CCr, RBC-DW and TBil, it also proved that intraoperative factors could affect the patients’ prognosis. High-preoperative RBC-DW could result from the decrease in erythropoietin (EPO) production and chronic heart failure, increasing the in-hospital mortality with AKI after cardiac surgery [43–45]. Elisabeth et al. also showed the high TBil level is prone to develop cholemic nephropathy, due to impair the structure of tubular epithelial plasma membranes and mitochondria [46, 47].

Postoperative MODS is a common final cause to death in critically ill patients, the mortality is approximately 54% [15, 31, 48, 49]. In most cases, patients with MODS are supported by continuous vasoactive agents or mechanism assistances to maintain vital signs [50]. The nature of MODS focuses on the crosstalk among different organs, damage from one organ could induce secondary injury for another organ, finally active a vicious circle [51]. Higher than 5% of postoperative patients will develop to MODS, especially LCOS and AKI-rH are combined [52]. In Table 4, intraoperative risk factors including ACPBT and cTVR were new predictors for postoperative MODS besides CCr, BUN/BCr and PD, we also found PD (OR 4.69) and cTVR (OR 3.69) were the high-risk factors for postoperative MODS. Researchers showed surgical intervention for severe tricuspid valve disease is only indicated in symptomatic patients, or who have severe comorbidities [53, 54]. Sharma et al. reported that right ventricular failure is related to nearly 40% kidney failure and has an increased mortality risk after TVR [55].

Currently, there are two classical prognostic models to predict in-hospital morality after heart valve surgery, the Society for Thoracic Surgeons (STS) score (https://www.sts.org/resources/risk-calculator) and European System for Cardiac Operative Risk Evaluation (EuroSCORE) II (http://www.euroscore.org/calc.html) [56, 57]. The limitations for these models were found as follows: the major endpoint is mortality, predictive accuracy only relies on preoperative factors, and model-based design of patients underwent CABG instead of heart valve surgery. Postoperative renal failure is the only common endpoint for STS score and PIRF model. The AUC of renal failure in the PIRF model is very close to that in STS score (0.780 vs. 0.787). But the PIRF model is a more simplified model with only 4 independent variables. Besides, the postoperative morbidity was defined quite differently by STS score and our PIRF model, the definition proposed by STS score applied to a wider range than PIRF model. Although a direct comparison between STS score and PIRF model could not be made, according to the official website of STS 2018 score, we find the AUC of postoperative morbidity in the STS score is lower than that in our PIRF model (STS score: 0.723 vs. PIRF model: LCOS 0.821, AKI-rH 0.780, MODS 0.774).

Basing on clinical observations and recent research results, important intraoperative factors can influence the prognosis of patients and the postoperative severe complications are associated with increased mortality. Nearly all variables contained in both STS score and EuroSCORE II are adopted in this study, besides, addition of the new variables of intraoperative risk factors were added. The endpoints in our study include postoperative sever complications, but not mortality. We proved that the intraoperative risk factors, especially ACPBT was the common independent risk factor for all endpoints (LCOS: OR 1.80; AKI-rH: OR 1.50; MODS: OR 1.71). Therefore, we provided a method for predicting severe complications morbidities after heart valve surgery by both preoperative and intraoperative factors are considered. Besides, the predictive power of PIRF models is more accurate and reliable compared with PRF models. Basing on this study, we provided a thought for conventional model to improve the predictive power for mortality and adjust treatment planning in time.

As a retrospective study, it also has some limitations. The sample size is limited by a single-center research and only focus on postoperative complications within 30 days. Therefore, it is necessary to validate the multivariable prognostic model by using a larger sample size from multiple centers and focus on long-term prognosis in the future.

## Conclusions

In this study, we consider both preoperative and intraoperative factors to predict severe complications morbidities after heart valve surgery, providing a further thought to improve the predictive power of conventional prognostic model for patients underwent heart valve surgery. After re-selected and re-determined relative risk variables, the PIRF model was more accurate and reliable by adding the intraoperative factors, which will help us adjust treatment planning in time to decrease mortality eventually.

## Data Availability

All data generated or analyzed during this study are included in this published article.

## Abbreviations

ACPBT: Auxiliary cardiopulmonary bypass time
AKI: Acute kidney injury
AKI-rH: Acute kidney injury requiring hemodialysis
ALB: Albumin
ALT: Alanine transaminase
AOT: Aortic occlusion time
APTT: Activated partial thromboplastin time
AUC: The area under the receiver-operator characteristic (ROC) curve
AVR: Aortic valve replacement
BCr: Blood creatinine
B.E.: Base excess indicate
BMI: Body mass index
BSA: Body surface area
BUA: Blood uric acid
BUN: Blood urea nitrogen
BUN/BCr: Blood urea nitrogen/blood creatinine
CABG: Coronary artery bypass graft surgery
CAD: Coronary artery disease
CAG: Coronary angiography
CCr: Creatinine clearance
CI: Cardiac index
CPB: Cardiaopulmonary bypass
CPBT: CPB time
cTVR: Combined tricuspid valve replacement
ECG: Electrocardiogram
ECMO: Extracorporeal membrane oxygenation
EF: Ejection fraction
ESR: Erythrocyte sedimentation rate
ESRD: End-stage renal disease
EuroSCORE: European system for cardiac operation risk evaluation
Fbg: Fibrinogen
GFR: Glomerular filtration rate
Hb: Hemoglobin
CF: Cardiac failure
HVD: Heart valve disease
IABP: Intra-aortic balloon pump
ICU: Intensive care unit
LASSO: Least absolute shrinkage selector operator
LCOS: Low cardiac output syndrome
LVD: Left ventricular dimension
MODS: Multiple organ dysfunction syndrome
MVP: Mitral valvuloplasty
MVR: Mitral valve replacement
NNECDSG: Northern New England cardiovascular disease study group
NYCSRE: New York’s cardiac surgery reporting system
PASP: Pulmonary artery systolic pressure
PiCCO: Pulse index contour cardiac output
PLT: Platelet
PIRF: Preoperative and intraoperative risk factors
PRF: Preoperative risk factors
PVD: Peripheral vascular disease
QMMI: Quality measurement and management initiative
RFA: Radiofrequency ablation
RBC: Red blood cell
RBC-DW: Red blood cell distribution width
ROC: Receiver operating characteristic curve
SaO_2_: Arterial oxygen saturation
STS: Society of thoracic surgeons
SvO_2_: Mixed venous oxygen saturation
TBil: Total bilirubin
TVP: Tricuspid valvuloplasty
TVR: Tricuspid valve replacement
UCG: Ultrasound cardiography
WBC: White blood cell
